# Determinants of the Incidence of Hand, Foot and Mouth Disease in China Using Geographically Weighted Regression Models

**DOI:** 10.1371/journal.pone.0038978

**Published:** 2012-06-18

**Authors:** Maogui Hu, Zhongjie Li, Jinfeng Wang, Lin Jia, Yilan Liao, Shengjie Lai, Yansha Guo, Dan Zhao, Weizhong Yang

**Affiliations:** 1 State Key Laboratory of Resources and Environmental Information System, Institute of Geographic Sciences and Natural Resources Research, Chinese Academy of Sciences, Beijing, China; 2 Key Laboratory of Infectious Disease Surveillance and Early-Warning, Chinese Center for Disease Control and Prevention, Beijing, China; 3 Chinese Research Academy of Environmental Sciences, Beijing, China; 4 Institute of Population Research, Peking University, Beijing, China; 5 Institute of Computing Technology, Chinese Academy of Sciences, Beijing, China; INSERM & Universite Pierre et Marie Curie, France

## Abstract

**Background:**

Over the past two decades, major epidemics of hand, foot, and mouth disease (HFMD) have occurred throughout most of the West-Pacific Region countries, causing thousands of deaths among children. However, few studies have examined potential determinants of the incidence of HFMD.

**Methods:**

Reported HFMD cases from 2912 counties in China were obtained for May 2008. The monthly HFMD cumulative incidence was calculated for children aged 9 years and younger. Child population density (CPD) and six climate factors (average-temperature [AT], average-minimum-temperature [AT_min_], average-maximum-temperature [AT_max_], average-temperature-difference [AT_diff_], average-relative-humidity [ARH], and monthly precipitation [MP]) were selected as potential explanatory variables for the study. Geographically weighted regression (GWR) models were used to explore the associations between the selected factors and HFMD incidence at county level.

**Results:**

There were 176,111 HFMD cases reported in the studied counties. The adjusted monthly cumulative incidence by county ranged from 0.26 cases per 100,000 children to 2549.00 per 100,000 children. For local univariate GWR models, the percentage of counties with statistical significance (*p*<0.05) between HFMD incidence and each of the seven factors were: CPD 84.3%, AT_max_ 54.9%, AT 57.8%, AT_min_ 61.2%, ARH 54.4%, MP 50.3%, and AT_diff_ 51.6%. The *R* ^2^ for the seven factors’ univariate GWR models are CPD 0.56, AT_max_ 0.53, AT 0.52, MP 0.51, AT_min_ 0.52, ARH 0.51, and AT_diff_ 0.51, respectively. CPD, MP, AT, ARH and AT_diff_ were further included in the multivariate GWR model, with *R* ^2^ 0.62, and all counties show statistically significant relationship.

**Conclusion:**

Child population density and climate factors are potential determinants of the HFMD incidence in most areas in China. The strength and direction of association between these factors and the incidence of HFDM is spatially heterogeneous at the local geographic level, and child population density has a greater influence on the incidence of HFMD than the climate factors.

## Introduction

Hand, foot and mouth disease (HFMD) is a common infectious disease, the main clinical symptoms of which include mouth ulcers and vesicles on the hands, feet, and mouth. The disease is caused by a group of non-polio enteroviruses, particularly those viruses belonging to Human Enterovirus species A (HEV-A). In most cases, the disease is mild and self-limiting, however, severe clinical presentations with neurological symptoms such as meningitis, encephalitis and polio-like paralysis, and pulmonary edema may occur, particularly among those aged 5 years and younger [1]. Currently, there is no vaccine or antiviral treatment specifically for HFMD.

Outbreaks of HFMD have been reported since the 1970s. Over the last decade, HFMD epidemic have increasingly occurred in countries of the Western Pacific Region, which were regarded as the most severely affected region by HFMD in the world, including Japan, Malaysia, and Singapore, Thailand, and China [2], [3], [4], [5]. The incidence of HFMD, particularly with deaths among children caused by severe complications, appears to be increasing across this region [6]. In 2009, for example, an epidemic in mainland China involved 1 155 525 cases, 13 810 severe cases and 353 deaths [1]. Therefore, HFMD has become an emerging public health concern in the affected countries, and a focus of increasing amounts of research.

In recent years, the epidemic, clinical, and pathogenetic features of HFMD have been studied extensively [2], [3], [4], [5], increasing our understanding of the distribution and severity of the disease. However, potential factors influencing the incidence of HFMD remain little understood. In this study, we explore the spatial association of HFMD incidence with several potential determinants (including child population density, average temperature, average minimum temperature, average maximum temperature, average temperature difference, average relative humidity, and monthly precipitation) to examine potential spatial variations in the assumed relationship between these factors and HFMD incidence.

## Methods

### Data

County-level HFMD data for May 2008 were obtained from the Chinese Center for Disease Control and Prevention. The total number of case records in May 2008 was 176 111, which is the highest number of monthly cases for the year. The case numbers of different counties vary substantially, from 0 to 2053 incident cases. According to the records obtained, 98% cases (172 542) were children aged 9 years and younger, with county-specific case numbers varying from 0 to 2020 incident child cases. In this research, we focus on the incidence of HFMD among children 9 years old and younger (0∼9). Hence, we calculate the HFMD cumulative incidence by adopting the count of HFMD cases aged 0∼9 years as the numerator, and the total population aged 0∼9 years as the denominator.

We also obtained data of monthly climate factors from the China Meteorological Data Sharing Service System. The data were collected from 727 meteorological stations in the whole of China. The six monthly climate factors in the study consisted of average temperature (AT), average minimum temperature (AT_min_), average maximum temperature (AT_max_), average temperature difference (AT_diff_), average relative humidity (ARH), and monthly precipitation (MP). Taking account of the county population density of children (CPD), a total of seven factors were used to explore the local determinants of HFMD incidence.

The geographic data were obtained from the Chinese National Administration of Surveying, Mapping and Geoinformation.

### Cumulative Incidence Inference

The HFMD dataset comprises the number of incident cases in each county. It reflects the occurrence of the disease in different regions, and guides the government’s allocation of medical resources. However, it cannot estimate the risk of contracting the disease because of the different population numbers among administrative regions, whose land areas are also different. To reduce the influence of population size, cumulative incidence (CI) was used to reflect the risk of contracting HFMD in each county. It measures the disease frequency during a period of time [7]. We used the crude CI of HFMD among children aged 9 years and younger in the *i* ^th^ county in May 2008, denoted as 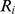. 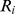 is the ratio of the number of cases 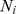 and the child population number 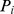 aged 9 years and younger. However, 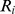 cannot be used to compare the disease risk between different counties directly because of random effects. Some counties reported 0 HFMD cases (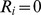) in May 2008, however, it could not be deduced that there is no risk of HFMD in these counties. In fact, they had HFMD cases in other months. The case number 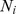 is a random variable. The observed number can be interpreted as just one realization of the random variable. Data collection error is another important source of variation. 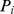 also affects the rate’s comparability. The minimum and maximum number of children (≤9 years) in the population of all counties is about 284 and 644 147, respectively. The larger 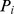 in the county, the more stable 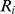 is. In contrast, an 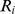 based on a small 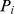 is less robust to data variation (for example, data collection error), than those calculated from larger 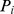. Therefore, a hierarchical bayes model, Besag, York, and Mollié (BYM) model, is adopted to reduce the spatial variance of CI [8]. The core idea to improve accuracy in the model is “borrowing strength” from other counties, whose accuracy is high. In the model, logarithm of the expected CI in the *i* ^th^ county 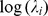 consists of three parts: the overall level of the disease risk 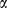, the correlated heterogeneity 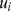 and the uncorrelated heterogeneity 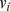, as following equation.
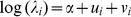
(1)


The uncorrelated heterogeneity 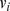 is an independent normal variable with mean 0 and variance 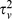. While for the correlated component 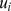, spatial correlation is involved in the model, which is defined by the intrinsic Gaussian auto-regression model [8], [9].
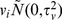
(2)

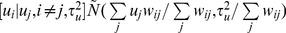
(3)where, 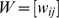 is the spatial adjacent matrix defining the connectivity between counties. 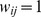 if the *i* ^th^ county and *j* ^th^ county is adjacent, otherwise 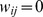. The parameters 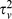 and 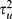 are the variability of 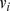 and 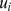 respectively. Gamma distribution is selected in the study as prior distribution where the hyper-parameters were drawn: 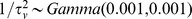 and 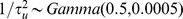. The model is solved by MCMC simulation in *WinBUGS* 1.4, and the length of burn-in sequence is 4 000. The convergence diagnostic is tested by the *boa* library in *R* 2.13 software.

### Climate Factors Interpolation

Climate factors are usually observed from spatially distributed meteorological stations. The data collected are site-specific. Inference is required to obtain climate factors (such as temperature and precipitation) for places where there are no meteorological stations. There are 727 meteorological stations distributed within the 2912 counties in the research. Interpolation is necessary to estimate each county’s mean value of the selected climate factors. By comparing the average interpolation accuracies from IDW, Kriging, and thin plate smoothing splines with “leave one out” validation scheme, the thin plate smoothing splines method was selected to estimate each county’s climate factors [10]. The expression of the thin plate smoothing splines method is as following:
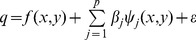
(4)where, 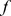 is an unknown smooth function to be estimated; 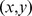 is spatial point’s coordinates; 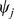 is a set of covariates, and 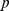 is number of covariates; 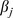 is a set of unknown parameters which will be estimated; 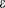 is normal distributed random errors with mean 0. Climate factors are usually affected by the local topography, so elevation was selected as the covariate to improve the precision of the estimates, while latitude and longitude were two independent position variables [11], [12]. Parameters were calculated by minimizing the generalized cross validation scheme.

### Geographically Weighted Regression

Multivariate regression model has been extensively applied in spatial epidemiology. A global multivariate regression model can capture the average strength and significance of statistical relationships between independent and dependent variables with just one equation for all data [13]. The relationship is assumed to be unchanged everywhere. However, it might hide potentially important local variations in the relationship. Geographically weighted regression (GWR) model allows for local spatial variation in the relationship between variables across the whole space [14], [15]. The form of GWR model we used is similar to global regression models; however, the parameters vary with spatial location:
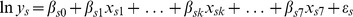
(5)where, 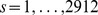 denotes spatial location of counties in mainland China; 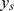 is the dependent variable CI*_s_*; seven independent variables 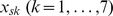, including child population density (logarithm transformed) and six climate factors, altogether are considered in the model; 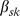 are local regression parameters to be estimated. Therefore, every county in our study area has a set of specific parameters to reflect the relationships between the CI and the seven independent variables.

Besides parameter localization, spatial autocorrelation is also carefully imbedded in the GWR model. According to Tobler’s first law of geography, everything is related to everything else, but near things are more related than distant things [16]. For a given county, variables from near counties are more important than those from far away counties. A weight matrix is adopted to represent the relative importance between counties. The weight value is a distance-decay function which is a Gaussian like “bell” shape function. The parameters are solved by the following matrix form, where 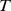 is the matrix transpose operation.
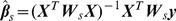
(6)

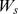
 is a diagonal weighting matrix as follows:



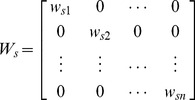
(7)The bandwidth or extent for a county to determine how many nearby counties should be included in the matrix is also a key point in the application because each county’s area is very different from others. Counties with sparse population densities often cover a large area, while counties with high population densities might have a small area. To make sure a sufficient number of counties are used to solve the local regression model, an adaptive kernel scheme is used to select the optimal number of neighboring counties. The optimal number is determined according to the Akaike Information Criterion (AIC) through an iterative optimization process [14]. AIC is one of the most appropriate indices for implementing the adaptive kernel technique to find the most appropriate number of neighbors of the regression county [15], [17]. The spatial radius to the regression county adapts for the density of counties at each regression location. A bi-square weighting function was selected to calculate the weight between counties,
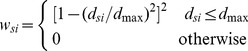
(8)where, *d*_max_ is the max distance from the *m* ^th^ farthest county to the county of interest (*m* is the selected optimal neighboring counties).

Both univariate and multivariate GWR models were built to explore the factors’ separate and combined explanatory effects. Factors entering into the multivariate GWR model were selected with the criteria of non-collinearity and AIC minimization. Furthermore, model significance was tested by variance analysis (*F* tests), and the significance of estimated local parameters was checked with pseudo *t* tests [14].

## Results

**Figure 1:**
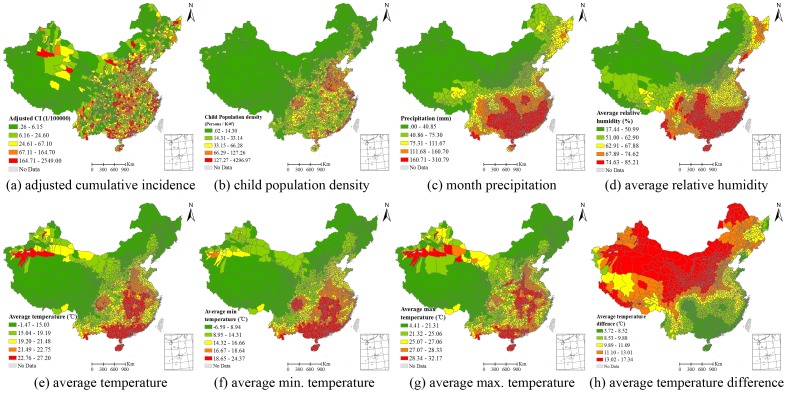
HFMD cumulative incidence of children (≤9 years old) and the spatial distribution of potential determinants.

The spatial distribution of the original monthly HFMD CI of children aged 0∼9 years in May 2008 was calculated from the county’s number of cases and the at-risk population. Among the 2912 counties, 468 counties reported no child HFMD cases (crude CI  = 0). To reduce uncertainty in the crude CI, a hierarchical Bayesian model was applied to adjust the rate. The county-specific CIs after adjustment are shown in Figure 1(a), and vary from 0.26 HFMD cases per 100 000 children to 2549.00 cases per 100 000 children with a median CI of 42.15 per 100 000 and a mean of 106.50 per 100 000. Simple comparisons of the original and adjusted CIs are listed in Table 1. No county has an adjusted CI of zero. The difference between the original CI and the adjusted CI is large in the counties with a sparse child population and a small number of cases. Counties with high HFMD CIs were mainly located in southern, eastern, mid- and northern China. Counties in west and north-east China had relatively low HFMD CIs. The global Moran’ *I* index of the adjusted CIs is 0.36 (*p*<0.01) indicating that there is relative strong positive spatial correlation.

**Table 1:** Summary of the original and adjusted HFMD cumulative incidences (1/100 000).

	Original CI	Adjusted CI
Minimum	0.00	0.26
1^st^ Quantile	7.32	9.06
Median	42.32	42.15
Mean	106.67	106.54
3^rd^ Quantile	132.00	131.03
Maximum	2555.34	2549.00

The spatial scope of the study area was very broad. Climate factors varied greatly from northwest to southeast China. Descriptive statistics for the climate and child population density data are presented in Table 2. The difference between the maximum and minimum average temperature is 28.67°C. The corresponding difference values of AT_min_, AT_max_ and AT_diff_ are 30.96°C, 27.76°C, and 13.62°C respectively. The variation in monthly precipitation is also very large. Some counties had more than 400 mm precipitation in May 2008; while in other counties, the precipitation is was less than 1 mm. This difference was reflected by the monthly average relative humidity index, whose difference between the maximum and minimum value is more than 60%. The child population number also varies greatly between counties, with a mean and standard deviation of 135.30 and 325.96 persons/Km^2^ respectively. The spatial distributions of child population density and the climate factors are displayed in Figure 1(b)–(h). High child population densities were mainly concentrated in eastern, northern, and southern China, while the child population densities in western and north-eastern China were smaller. Precipitation and temperature show a similar trend across China in May, decreasing from south-eastern to north-western China, which is opposite to the observed variation in temperature difference.

**Table 2:** Descriptive statistics of climate factors and child population density for all counties in May 2008, China.

	AT (°C)	AT_min_ (°C)	AT_max_ (°C)	AT_diff_ (°C)	ARH (%)	MP (mm)	CPD (person/km^2^)
Minimum	−1.47	−6.59	4.41	3.72	17.44	0.00	0.02
Mean	18.89	13.96	24.66	10.70	62.42	103.27	135.30
Maximum	27.20	24.37	32.17	17.34	85.21	310.79	4297.00
Standard Deviation	5.01	5.83	4.56	2.47	13.35	70.28	325.96

AT: average temperature; ARH: average relative humidity; MP: month precipitation; CPD: child population density.

**Figure 2:**
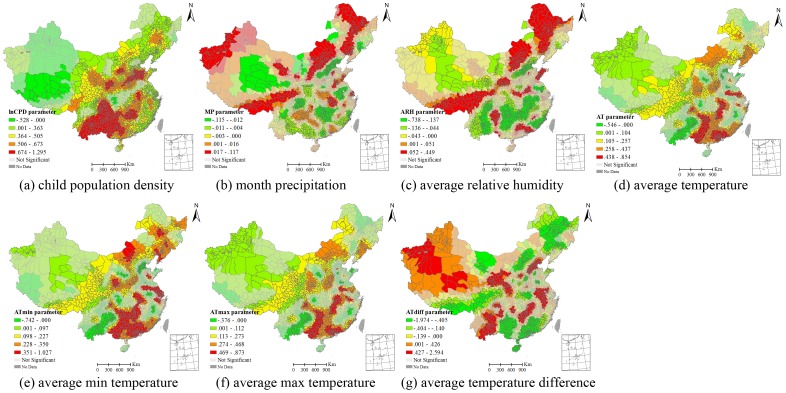
The spatial distribution on the local relationship between HFMD incidence, child population density, and six climate factors at county level in May 2008, China.

Univariate GWR models were built to test for significant relationships between HFMD CI and each potential explanatory factor. The coefficients of determination (adjusted) for AT, AT_min_, AT_max_, AT_diff_ and ARH are 0.52, 0.52, 0.53, 0.51 and 0.51 respectively (Table 3). The corresponding values of MP and CPD are 0.51 and 0.56 respectively. The GWR model with CPD as a unique explanatory variable has the lowest AIC value and the largest coefficient of determination among all the univariate models. The statistical significance of the regression parameters was checked using the pseudo *t* test. The percentages of counties with significant relationships (*p*<0.05) between CI and the factors (from largest to smallest percentage) are: CPD 84.3%, AT_min_ 61.2%, AT 57.8%, AT_max_ 54.9%, ARH 54.4%, AT_diff_ 51.8%, and MP 50.3%. Among counties, the directions of the significant relationships were not the same, even for the same factor. The percentages of counties with significantly positive relationships are: CPD 83.4%, AT_min_ 53.8%, AT 52.0%, AT_max_ 50.3%, MP 24.6%, ARH 19.7%, and AT_diff_ 25.1%. The spatial distribution of the direction and strength of the relationships between the HFMD CI and the seven factors is displayed in Figure 2. The model residuals’ global Moran’s I index are CPD 0.058, AT 0.093, AT_min_ 0.093, AT_max_ 0.091, AT_diff_ 0.096, MP 0.095, and ARH 0.094 (*p*<0.01) respectively. Although the model residuals’ spatial correlation is much smaller than the CI’s spatial correlation whose Moran’s *I* is 0.36, more effective variates would be helpful to capture the CI’s spatial variation.

**Table 3:** Summary of univariate GWR models for different factors.

	*R* ^2^	Significantly related counties		
		*p*<0.05	+	−
AT	0.52	57.8%	52.0%	5.8%
AT_min_	0.52	61.2%	53.8%	7.4%
AT_max_	0.53	54.9%	50.3%	4.7%
AT_diff_	0.51	51.8%	25.1%	26.6%
ARH	0.51	54.5%	19.7%	34.8%
MP	0.51	50.3%	24.6%	25.7%
CPD	0.56	84.3%	83.4%	0.9%

AT: average temperature; ARH: average relative humidity; MP: month precipitation; CPD: child population density.

**Figure 3:**
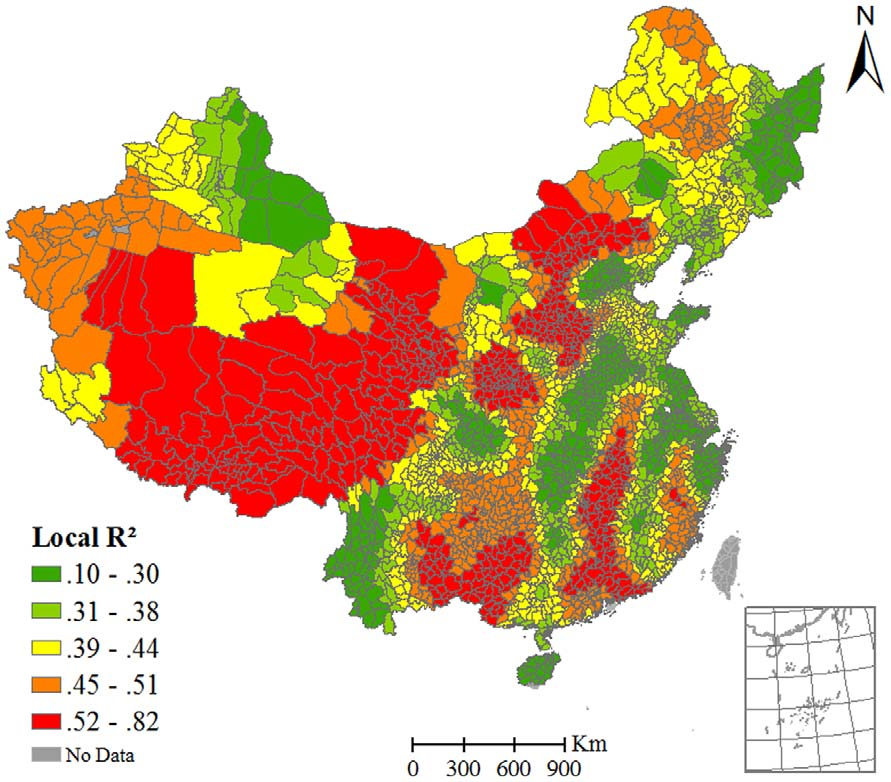
Local *R* ^2^ derived from multivariate GWR model.

All of the explanatory variables were further tested using a multivariate GWR model. Five variables (CPD, AT, ARH, MP and AT_diff_) were entered into the model under the criteria of non-collinearity and AIC minimization. The model’s coefficient of determination was 0.62, which is larger than that of all the univariate GWR models. Both AIC and ANOVA analysis (*F* test) show that the improvement is statistically significant. The global Moran’s *I* index of the regression residual is 0.03 (*p*<0.01), indicating that there is very weak positive spatial correlation. Thus the multivariate GWR model captures the CI’s variation very well. The local coefficients of determination at county level derived from the multivariate GWR model are shown in Figure 3. The spatial distribution of the local coefficients of determination demonstrates the combined statistical effect of the five explanatory variables on the HFMD CI. The coefficients of determination vary from 0.10 to 0.82 across the region. All of the local models are statistically significant (*p*<0.05). Summary of the relationship directions shows that CPDs of 92.6% counties are positively related with the HFMD CIs. It is much higher than all the four climate factors (AT 61.1%, AT_diff_ 49.2%, MP 62.5, and ARH 39.5%). Most of the directions in the multivariate GWR model are the same with that in the univariate models (Table 4). However, there are also a considerable number of the relationship directions changed between the univariate models and multivariate models. With the pseudo *t* test, CPDs of 60.06% counties are significantly related with the HFMD CIs in both univariate and multivariate model. The percentages of counties with significant relationships between CI and the climate factors in both univariate and multivariate models are: ARH 31.5%, AT_diff_ 26.2%, MP 25.2%, and AT 21.6%. Therefore, the proportion of CPD is also the highest one in the five factors. There are some counties where the relationships are significant only in the univariate model or multivariate model. There are also some counties where the relationships are not significant in neither models.

**Table 4:** Directions of relationship summary of the multivariate GWR model and univariate models.

	Directions of relationship in the multi-GWR model			Same directions in both uni- and multi-GWR models		Significant test of the relationships in uni- and multi-GWR models (*p*<0.05)			
	+	−	sig. (*p*<0.05)	+	−	both models	only uni-GWR	only multi-GWR	neither model
CPD	92.6%	7.4%	64.6%	91.6%	4.4%	60.6%	23.7%	3.9%	11.8%
AT	61.1%	38.9%	37.8%	54.3%	12.0%	21.6%	36.2%	16.2%	26.0%
AT_diff_	49.2%	50.8%	42.4%	29.1%	33.9%	26.2%	25.6%	16.2%	32.0%
MP	62.5%	37.5%	46.4%	37.2%	25%	25.2%	25.0%	21.2%	28.6%
ARH	39.5%	60.5%	48.8%	24.8%	44.9%	31.5%	23.0%	17.3%	28.2%

multi-GWR: multivariate GWR; uni-GWR: univariate GWR.

## Discussion

Child population density and climate factors were the potential determinants of HFMD incidence in most areas of China. Furthermore, the strength and direction of association between these factors and HFDM incidence were obviously spatially heterogeneous at the local geographic level. In some areas, the child population density and climate factors were not significantly related to the incidence of HFMD, while in the other areas these factors were positively related, or had an inverse association with HFMD incidence, with different strengths. We found that child population density exerted greater influence on HFMD incidence than the climate factors.

The strong seasonality of HFMD incidence and epidemics has been demonstrated in many affected countries and regions, for example, in mainland China epidemic peaks occurred in spring and early summer [18], the incidence of HFMD is highest in summer in Taiwan (China) [2], and outbreaks occurred in a cyclical pattern every 3 years in Malaysia [19]. Other studies have also assessed the influence of climate on the incidence of HFMD. Under a regression model, mean temperature, relative humidity, and wind speed were positively related to HFMD incidence rates in Hong Kong, where relative humidity was the most influential factor and wind speed was the least [20]. A similar relationship was found in Singapore, where weekly mean temperature and cumulated rainfall were significantly associated with HFMD incidence at a time lag of 1–2 weeks [21]. Under the analysis of an S-BME spatiotemporal model, the number of HFMD cases showed a close relationship to monthly precipitation in mainland China [22]. Similar to these studies, our study revealed that climate factors are associated with the incidence of HFMD. What’s more, we further demonstrated a spatial variation in the association between HFMD incidence and climate factors at the small geographic level of county, which has not been demonstrated previously. Child population density was also found to affect the incidence of HFMD in most of the study counties, which is consistent with the findings of another study recently conducted at the prefecture level (one higher administrative level than county) by other Chinese researchers [18]. By adopting a smaller geographic scope we further proved the spatial heterogeneity of the association between child population and HFMD incidence. There are two reasons that we select one month (May) rather than a year in 2008 to research the heterogeneous relationships between HFMD incidence and potential factors. On one side, the HFMD case number in May 2008 is the largest one in all the twelve months in 2008. It is the most typical month to reveal the relationships. On the other side, the heterogeneous relationships may be hidden or smoothed due to yearly mean value of potential factors.

We found that child population density alone could explain 56% of the variance of HFMD incidence, while the climate factors could explain about 52%. From this point of view, child population density seems more significantly related to the disease incidence rather than the climate factors. Among the 2912 study counties, the local GWR models’ statistical tests show that no factor is significantly related to the HFMD CI in all counties. However, the child population density factor had the largest percentage of significantly related (to HFMD CI) counties, and in most of these counties, population density was positively related to the HFMD CI. Besides child population density, three temperature-related factors (AT, AT_min_ and AT_max_) had positive relationships with the disease in more than 50% of the significantly related counties. In contrast, for average relative humidity, monthly precipitation and average temperature difference, more counties are negatively related to the HFMD CI than positively related. In addition, our study shows that the combination of child population density, monthly precipitation, average temperature, average temperature difference and average relative humidity could explain HFMD incidence more than any single factor, demonstrating the interactive effects among these factors on the disease incidence.

There are two possible reasons for the spatial heterogeneity of the relationship between the climatic factors and population density with HFMD incidence at the local level. First, weather condition is a synthesis of multiple climatic processes, and different combinations of climate factors may generate different climate types, which in turn would have unique influences on HFMD infection and transmission. For example, some combinations would favor virus survival, reproduction, and transmission [23], while others would limit children’s outdoor activity and would prevent viral transmission among the community [24], [25]. Second, other conditions unaccounted for in our study, such as population immunity to HFMD, public health measures taken by local health departments, and personal and environmental hygiene, may also contribute to the occurrence, transmission and spread of HFMD among the community, childcare centers, kindergartens, and preschools [3], [26], [27]. These factors are potential covariates influencing the association between child population density, climate factors, and the incidence of HFMD.

In spatial regression models, a global model is generally used to examine the relationship between disease risk and potential explanatory factors, which is based on the assumption that the relationship is a stationary spatial process [20], [21]. For a small and homogenous region of interest, it is reasonable to assume that the explanatory factors would not change significantly across the whole region, and the relationship between HFMD incidence and the potential factors would also be unchanged. However, the topography, climate, and population distribution change greatly when it comes to a large region like China with a territory over 9.6 million square kilometers. According to Zheng *et al’s* (2010) climate regionalization scheme, there are 12 temperature zones, 24 moisture regions, and 56 climatic sub-regions in China [28]. It would be difficult to keep the spatial stationarity assumption in such a complicated area. The effect of the factors of interest on HFMD would be more similar in local regions with similar conditions, while the effect would be more different in local regions whose conditions differ greatly. GWR models consider spatial heterogeneity by separating the large heterogeneous region into small local regions. Only nearby counties are included in the local regression, and every included county is given a weight according to its spatial distance to the destination county. In this study, GWR models were successfully used to explore the local climate and population distribution factors effects on HFMD incidence at the county level, which demonstrates that GWR models can be used to geographically differentiate the relationships between diseases and their explanatory factors.

The underreporting of HFMD cases in clinics and hospitals is a potential limitation of our study. Some HFMD cases do not seek health care because their symptoms are mild, or they are asymptomatic. Also, even though standard diagnosis criteria for HFMD have been issued by the Chinese Ministry of Health, discrepancies in clinical diagnosis awareness and capacity exists in the clinics and hospitals among different regions (such as in developed versus underdeveloped areas). Regional differences in the reporting of HFMD cases may influence our study findings to a certain extent.

In summary, HFMD, a wide-spread infectious disease in China, is found to be heterogeneously related to climate factors and child population density distributed at the county level throughout the country. Our findings may assist in the risk assessment for HFMD epidemic in local areas, and guide local public health institutes to rationally allocate public health resources and improve their preparedness for an outbreak according to region-specific conditions.
